# Emergency department and hospital admissions among people with dementia living at home or in nursing homes: results of the European *RightTimePlaceCare* project on their frequency, associated factors and costs

**DOI:** 10.1186/s12877-020-01835-x

**Published:** 2020-11-05

**Authors:** F. Javier Afonso-Argilés, Gabriele Meyer, Astrid Stephan, Mercè Comas, Ansgar Wübker, Helena Leino-Kilpi, Connie Lethin, Kai Saks, Maria Soto-Martin, Caroline Sutcliffe, Hilde Verbeek, Adelaida Zabalegui, Anna Renom-Guiteras, on behalf of the RightTimePlaceCare Consortium, Gabriele Meyer, Astrid Stephan, Anna Renom-Guiteras, Dirk Sauerland, Ansgar Wübker, Patrick Bremer, Jan P. H. Hamers, Basema Afram, Hanneke C. Beerens, Michel H. C. Bleijlevens, Hilde Verbeek, Sandra M. G. Zwakhalen, Dirk Ruwaard, Ton Ambergen, Ingalill Rahm Hallberg, Ulla Melin Emilsson, Staffan Karlsson, Christina Bokberg, Connie Lethin, David Challis, Caroline Sutcliffe, David Jolley, Sue Tucker, Ian Bowns, Brenda Roe, Alistair Burns, Helena Leino-Kilpi, Jaana Koskenniemi, Riitta Suhonen, Matti Viitanen, Seija Arve, Minna Stolt, Maija Hupli, Kai Saks, Ene-Margit Tiit, Jelena Leibur, Katrin Raamat, Angelika Armolik, Teija Tuula Marjatta Toivari, Adelaida Zabalegui, Montserrat Navarro, Esther Cabrera, Ester Risco, Carme Alvira, Marta Farré, Susana Miguel, Maria Soto, Agathe Milhet, Sandrine Sourdet, Sophie Gillette, Bruno Vellas

**Affiliations:** 1 Department of Geriatric Medicine and Palliative Care. Badalona Serveis Assistencials, Barcelona, Spain; 2 Universitat Autònoma de Barcelona, Barcelona, Spain; 3 Department of Nursing Science, Witten/Herdecke University, Witten, Germany; 4 Institute of Health and Nursing Sciences, Medical Faculty, Martin Luther University Halle-Wittenberg, Halle (Saale), Germany; 5 Department of Epidemiology and Evaluation. Hospital del Mar, Parc de Salut Mar, Barcelona, Spain; 6 Member of the Health Services Research on Chronic Patients Network (REDISSEC), Madrid, Spain; 7 RWI – Leibniz-Institute for Economic Research, Leibniz Science Campus Ruhr and RUB, Essen, Germany; 8 Department of Nursing Science and Nurse Director, Turku University Hospital, University of Turku, Turku, Finland; 9 Department of Health Sciences. Faculty of Medicine, Lund University, SE-221 00 Lund, Sweden; 10 Clinical Memory Research Unit. Department of Clinical Sciences, Lund University, SE-221 00 Lund, Sweden; 11 Department of Internal Medicine, University of Tartu, Tartu, Estonia; 12 Department of Geriatric Medicine, Gerontopole, Alzheimer Disease Research Center, Inserm UMR 1027, University Hospital of Toulouse, Toulouse, France; 13 School of Health Sciences, University of Manchester, Manchester, UK; 14 Department of Health Services Research, Care and Public Health Research Institute, Maastricht University, Maastricht, The Netherlands; 15 Living Lab in Ageing and Long-Term Care, Maastricht, The Netherlands; 16 Hospital Clinic de Barcelona, Barcelona, Spain; 17 School of Medicine, Universitat de Barcelona, Barcelona, Spain; 18 Department of Geriatric Medicine, Parc de Salut Mar, Barcelona, Spain

**Keywords:** Aged, Dementia, Geriatric syndrome, Home care, Hospitalisation, Nursing home

## Abstract

**Background:**

Evidence is lacking on the differences between hospitalisation of people with dementia living in nursing homes and those living in the community. The objectives of this study were: 1) to describe the frequency of hospital admission among people with dementia in eight European countries living in nursing homes or in the community, 2) to examine the factors associated with hospitalisation in each setting, and 3) to evaluate the costs associated with it.

**Methods:**

The present study is a secondary data analysis of the *RightTimePlaceCare* European project. A cross-sectional survey was conducted with data collected from people with dementia living at home or who had been admitted to a nursing home in the last 3 months, as well as from their caregivers. Data on hospital admissions at 3 months, cognitive and functional status, neuropsychiatric symptoms, comorbidity, polypharmacy, caregiver burden, nutritional status, and falls were assessed using validated instruments. Multivariate regression models were used to investigate the factors associated with hospital admission for each setting. Costs were estimated by multiplying quantities of resources used with the unit cost of each resource and inflated to the year 2019.

**Results:**

The study sample comprised 1700 people with dementia living in the community and nursing homes. Within 3 months, 13.8 and 18.5% of people living in nursing homes and home care, respectively, experienced ≥1 hospital admission. In the nursing home setting, only polypharmacy was associated with a higher chance of hospital admission, while in the home care setting, unintentional weight loss, polypharmacy, falls, and more severe caregiver burden were associated with hospital admission. Overall, the estimated average costs per person with dementia/year among participants living in a nursing home were lower than those receiving home care.

**Conclusion:**

Admission to hospital is frequent among people with dementia, especially among those living in the community, and seems to impose a remarkable economic burden. The identification and establishment of an individualised care plan for those people with dementia with polypharmacy in nursing homes, and those with involuntary weight loss, accidental falls, polypharmacy and higher caregiver burden in the home care setting, might help preventing unnecessary hospital admissions.

## Background

Dementia is a major cause of disability and dependency among older people worldwide and has a psychological, social and economic impact on the persons affected, their families and caregivers, and the society [1]. People with dementia have increasing needs for care and supervision throughout the course of their disease, usually leading to the need for support from relatives or other informal caregivers and, when the needs of people with dementia are increased, many of them need to receive care in nursing homes (NH) [2].

Admission to hospital due to acute medical problems, including the admission to the emergency departments, is one of the events that people with dementia may experience throughout the course of the disease, irrespectively of whether they live at their own home or at NH. Despite the benefits of receiving specialized care, multiple negative health outcomes have been associated with the admission to hospital of older people in general and people with dementia in particular, including high risk of delirium [3], functional decline [4], fall-related injuries [5], nosocomial infections, pressure ulcers [6], and an elevated risk of mortality [7, 8].

In a recent systematic review of the literature, Shepherd et al. (2019) concluded that people with dementia are more frequently admitted to hospital than those without dementia, and identified hospitalisation rates in people with dementia between 0.37 and 1.26/person-year in high-quality studies [9]. However, studies on people with dementia that predominantly resided in long-term nursing care facilities were excluded from this review. Maxwell et al. (2015) reported an annual hospitalisation rate of 0.10 per person-year among people with dementia living in NH [10].

In their meta-analysis, Shepherd et al. (2019) found strong evidence that multimorbidity, polypharmacy, and lower functional ability are associated with hospital admission, and strong evidence that dementia severity alone is not associated [9]. However, there is a lack of studies reporting on factors associated with hospitalisation among people with dementia living in NH.

Geriatric syndromes are known to predict an increased likelihood of hospitalisation, affect quality of life [11], increase the use and cost of health care, and raise overall mortality among the general older population [12, 13]. However, relevant geriatric syndromes such as malnutrition or falls have not been evaluated as potential factors associated with hospital admissions in the studies involving people with dementia [9]. Furthermore, even if caregivers of people with dementia have a crucial role in the care and resource use in this population, only few studies have analyzed the effects that their psychological wellbeing may have on the decisions of hospital admission of these people [14].

Even if the economic cost of dementia has been widely evaluated in recent years [15], only few studies have provided a detailed description of the costs of hospital admission among people with dementia taking their living situations into consideration [16]. This information may be helpful for the planning of the provisions of care.

Thus, there is a lack of studies that describe and compare the frequency of hospital admission in both the community and NH settings, investigate and compare the factors associated with hospital admission in both settings, and provide information on its costs.

### Aim

The aims of the present study were: 1) to describe the frequency of hospital admissions (including Emergency Department), among people with dementia from eight European countries living in the community and those living in nursing homes; 2) to examine the factors associated with hospital admissions among people with dementia living in the community and those living in nursing homes settings, separately; 3) to evaluate the costs associated with hospital admissions according to the different living situations.

## Methods

### Design

This study is a secondary analysis of a large European research project called *‘RightTimePlaceCare*’ (RTPC). The RTPC survey was a longitudinal prospective study, conducted in eight European countries (Estonia, Finland, France, Germany, Netherlands, Spain, Sweden, and the United Kingdom). The project aimed to improve health and social care services for European citizens with dementia and focused on the transition from professional home care towards institutional nursing care. Further details are published elsewhere [17].

### Participants

The RTPC survey comprised two groups of participants: 1) People with dementia newly admitted to NH (i.e. within one to 3 months after admission) and their informal caregivers; 2) People with dementia who received professional home care and were at risk of institutionalization (as judged by a professional responsible for their care) along with their informal caregivers. Informal caregivers could include spouses/partners, other family members, relatives, friends, neighbours or other unpaid individuals within their social network [18]. The number of informal caregivers was limited to one main informal caregiver per person with dementia, defined as the person who was most involved in care for the people with dementia. For people living in NH, the next of kin or significant other was included, being the person closest to the person with dementia (spouse, children, grandchildren, other relatives, or friends) and who was most involved in decisions about their care.

Inclusion criteria for people with dementia were if they (1) had a primary diagnosis of dementia as diagnosed by an expert (e.g. physician, psychiatrist, neurologist, geriatrician, or general practitioner, depending on countries’ diagnostic procedures) and recorded in the medical records; (2) scored 24 points or lower on the Standardized Mini Mental State Examination (S-MMSE) [19]; and (3) had an informal caregiver who visited the people with dementia at least twice a month. People with dementia were excluded when they (1) were younger than 65 years; (2) had a primary psychiatric diagnosis or Korsakoff syndrome; and, for the group recently admitted to NH, (3) were only temporarily resident in the NH (e.g. rehabilitation, respite of the informal caregiver) with the intention of moving back home.

### Procedures

Each country obtained ethical approval from a country specific legal authority for research on human beings (for example an ethical committee specialized in medical or nursing science) to conduct the study in accordance with their national standards and regulations. Country-specific consent procedures were followed.

Data were collected by trained interviewers at baseline between November 2010 and April 2012 and follow-up interviews were conducted after 3 months [17]. All interviewers were professionals in health or social care or medical/nursing/social care students with practical experience and at least a Bachelor’s degree.

### Measures

#### Sample characteristics

At baseline, information on the age, sex, country and living situation (i.e. home care (HC) vs nursing home (NH)) of the people with dementia was collected. Cognitive status was assessed with the S-MMSE (range 0–30), with higher scores indicating less cognitive impairment [19]. The disease severity was defined as mild (S-MMSE > 21), moderate (10–21), or severe (< 10) [20]. Participants who were unable to perform the S-MMSE due to cognitive or functional impairment were placed into the “severe dementia” group. Medical history of past and current clinical conditions were recorded based on the Charlson Comorbidity Index (CCI) (range 0–37), with higher scores indicating more clinical conditions and a score > 2 indicating moderate comorbidity [21]. Independence in activities of daily living (ADL) was measured by the Katz index (range 0–6); we categorised dependency as mild (Katz index 5–6), moderate (2–4), or severe (< 2) [22]. Behavioural and psychiatric symptoms in dementia were evaluated with the Neuropsychiatric Inventory Questionnaire (NPI-Q). The NPI-Q comprises 12 domains (e.g. agitation and aggression, delusions, hallucinations) with 2 scores reported for each domain: (1) presence of symptoms and (2) severity on a 0–3 scale (0 = none, 1 = mild, 2 = moderate, 3 = severe; range 0–36), with higher scores indicating more (severe) symptoms [23].

Furthermore, several quality of care indicators based on recent literature [24] were assessed at baseline: nutritional status (one item question ‘did the person experience a weight loss of 4% or more of his/her weight in the past year?’) [25, 26], polypharmacy (≥5 drugs) [27] and the occurrence of falls during the preceding 3 months. Fall was defined as “an unexpected event in which the participant comes to rest on the ground, floor, or lower level” [28].

With regard to the informal caregivers, data on their age, sex and relationship with the person with dementia were collected. In addition, caregiver burden was measured using the Zarit Burden Interview (ZBI) [29]. Informal caregivers responded to 22 statements relating to the care of their relative with dementia using a Likert scale scoring system which ranged from “Never” to “Nearly Always”. Total scores range from 0 to 88, with a higher score indicating higher perceived burden; three severity groups were defined: little or no burden (ZBI < 21), mild burden (ZBI ≥21–40), or moderate to severe burden (ZBI > 40) [30].

In the HC setting, information on independence in ADL, behavioural and psychiatric symptoms in dementia and quality of care indicators was asked to the informal caregivers of the people with dementia, while in the NH setting, this information was asked to the formal caregivers, as they were considered best informed proxies.

All measurement instruments were selected based on their psychometric properties (validity, reliability), clinical utility and appropriateness for the target settings and population [17].

#### Information on emergency department and hospital admissions

The Resource Utilization in Dementia (RUD) instrument was used to determine the health care resources used for people with dementia [31]. The RUD instrument captures medical care resources use (e.g. medication, inpatient and outpatient care), community care services (e.g. home care, transportation, meals on wheels, day care), special accommodation (e.g. nursing home) and time spent with caring activities by informal caregivers. Information on hospital visits since the baseline interview was gathered using an adapted version of the RUD instrument in the follow-up assessment that included the two concepts Hospital Admission (HA) and Emergency Department Admissions (EDA). In the HC setting, this information was asked to the informal caregivers, while in the NH setting, this information was asked to the formal caregivers, as they were considered best informed proxies. With regard to HA, caregivers were asked: “since the last visit, was the person with dementia admitted to a hospital (for more than 24 hours)?”; “how many times was your relative/the resident hospitalised?”; “for each hospitalisation, please provide the diagnosis or reasons for hospitalisation”; “for each hospitalisation, please specify the total number of nights spent in each type of ward”. The following types of ward were asked about: geriatric ward, surgery ward, internal medicine ward, psychiatry ward and other hospital stays. If the answer “other” was given, the caregiver was asked to specify the type of ward by means of an open question. With regard to EDA, caregivers were asked: “Since the last visit, did the person with dementia receive care in an accident and emergency hospital department (for less than 24 hours)?”; and if yes, “how many times”? Participants with at least one episode of HA or EDA were considered to have experienced a hospital visit.

#### Estimate of cost for emergency department and hospital admissions

While the costs of dementia were previously measured from a societal perspective in the RTPC project [33–35], our study focused on the assessment of direct medical costs generated by EDA and HA and calculated as average costs per capita and year in Euros (€). In the absence of valid and comparable unit costs for all participating countries, a common price vector based mainly on Swedish sources was used for all countries [33, 36]. For HA, the length of stay in each medical department was taken into account in the measurement. In the case of fees not being available for daily stay in departments other than internal medicine, geriatrics or surgery it was decided to assume a cost per day similar to the daily expense in the department of geriatrics. In addition, we calculated the average costs per capita and year specifically among those people with dementia who had suffered ≥1 hospital admission. All costs were inflated to the year 2019 and currency conversion adjustments based on 2019 exchange rates were made for the non-EURO countries. Cost components, unit costs, the international adjustment schemes and their sources are shown in Table 1 [32, 36, 37].

**Table 1:** Unit costs and international price adjustments (inflated to the year 2019^a^)

	Estonia	Finland	France	Germany	Netherlands	Spain	Sweden	UK
**International price adjustment**								
Exchange rates; local currency / Euro	1.00	1.00	1.00	1.00	1.00	1.00	10.59	0.87
**Medical Care (Inpatient care)**								
Costs^b^ per day hospital (geriatric)	92.14	318.60	313.82	293.41	280.82	162.22	335.25	256.98
Costs per day hospital (internal medicine)	101.27	350.18	344.92	322.50	308.64	179.13	368.50	282.45
Costs per day hospital (surgery)	148.87	514.74	507.01	474.05	453.69	263.31	541.63	415.19
Costs Emergency room visit	63.06	218.14	214.86	200.89	192.27	111.59	229.54	175.94

^a^ Last available official statistics by the Organisation for Economic Co-operation and Development (OECD). Prices, purchasing power parities (PPP) and Exchange rates [32]

^b^Costs are expressed in Euros

### Statistical analyses

The characteristics of people with dementia and their informal caregivers as well as the HA and EDA episodes are described using means and standard deviations (SD) for continuous variables with normal distribution, median, first and third quarter for continuous variables without normal distribution, and percentages for discrete variables. The assessment of the hospitalisation rates was made taking into account the estimated annual emergency department and hospital admissions, respectively, in addition to the total number of people with dementia participating in the study.

Univariate analyses were used to evaluate the independent variables (CCI, S-MMSE, Katz Index, NPI-Q scores, ZBI, weight loss, polypharmacy, falls and setting) potentially associated with the occurrence of a Hospital Admission (at least one episode of HA or EDA). Variables were chosen after literature-based team discussion. Mann-Whitney’s U test was used for continuous variables, as the distribution of the variables was not normal and the number of cases was small. Fisher’s exact test was preferred when the software was available to calculate it (all categorical variables except country), even though the approximation using the Chi-square test was good because of sufficient sample size in all categories [38]. All variables were included in a logistic regression model. The different independent variables were tested on multicollinearity in order to identify potential interactions between them.

All analyses considered a significance level α of 0.05 (two-sided) and were conducted with IBM SPSS Statistics version 25 (IBM Corporation, Chicago, IL).

## Results

### Sample characteristics

The total RTPC sample at baseline comprised 2014 dyads of people with dementia and their informal caregivers. For the present study, only participants with data available on EDA and HA were included. Thus, the study sample comprised 1700 people with dementia and their informal caregivers (*n* = 1054 (62%) from the HC setting and *n* = 646 (38%) from the NH setting).

The mean age of the study sample was 82.8 years (standard deviation (SD) 6.5), and 68.5% were women. The characteristics of the study participants (people with dementia and informal caregivers) are summarized in Table 2 and Additional file 1, respectively.

**Table 2:** Baseline characteristics of the people with dementia, by setting

Variables	Nursing Home (***n*** = 646)	Home Care (***n*** = 1054)	Overall (***n*** = 1700)
Age (years), mean (SD)	83.8 (6.5)	82.1 (6.5)	82.8 (6.5)
Women, n (%)	486 (75.2%)	679 (64.4%)	1165 (68.5%)
S- MMSE (range 0–30)^a^,^b^, median (Q1;Q3)	13 (8;17)	15 (10;20)	14 (9;19)
Missing values	128	130	258
Katz Index (range 0–6)^a^, median (Q1;Q3)	2 (1;4)	3 (2;5)	3 (1;5)
Missing values	2	11	13
Charlson Comorbidity Index (range 0–37)^a^, median (Q1;Q3)	2 (1;3)	2 (1;3)	2 (1;3)
Severity of neuropsychiatric symptoms (range 0–36)^a^, median (Q1;Q3)	5.5 (3;10)	8 (4;13)	7 (3;12)
Zarit Burden Interview (range 0–88)^a^, median (Q1;Q3)	21 (13;33)	31 (20.9;43)	27 (17;39.2)
Missing values	3	3	6
Weight loss (≥4%, yes), n (%)	81 (13.0%)	211 (20.6%)	292 (17.7%)
Missing values	25	28	53
Polypharmacy (≥5 drugs, yes), n (%)	440 (68.1%)	604 (57.3%)	1044 (61.4%)
Mean number of drugs (SD)	6.0 (3.1) [6]	5.3 (3.1) [5]	5.5 (3.1) [5]
Falls in preceding 3 months (baseline)	172 (26.7%)	215 (20.4%)	387 (22.8%)
Missing values	3	1	4
Country, n (%)			
United Kingdom	55 (8.5%)	60 (5.7%)	115 (6.8%)
Estonia	80 (12.4%)	145 (13.8%)	225 (13.2%)
Finland	111 (17.2%)	157 (14.9%)	268 (15.8%)
France	35 (5.4%)	151 (14.3%)	186 (10.9%)
Germany	99 (15.3%)	91 (8.6%)	190 (11.2%)
Netherlands	104 (16.1%)	165 (15.7%)	269 (15.8%)
Spain	85 (13.2%)	155 (14.7%)	240 (14.1%)
Sweden	77 (11.9%)	130 (12.3%)	207 (12.2%)

Missing values declared when present

*SD* Standard Deviation, *Q1* First quartile, *Q3* Third quartile

^a^ Underlined scores indicate the direction of a better outcome

^b^Only participants with scores of lower than 24 points were included

### Emergency department or hospital admissions

Table 3 describes the frequencies of EDA and HA in both settings as well as, for HA, the frequencies of admission and the average length of stay in days in the different medical departments evaluated.

**Table 3:** Description of Emergency Department Admissions (EDA) and Hospital Admission (HA), by setting

	Nursing Home (***n*** = 646)	Home Care (***n*** = 1054)	Overall (***n*** = 1700)	***p***_value****
PwD* with at least 1 EDA or 1 HA**, n (%)	89 (13.8%)	195 (18.5%)	284 (16.7%)	0.011
PwD with at least 1 EDA (with < 24 h stay), n (%)	48 (7.4%)	123 (11.7%)	171 (10.1%)	0.005
PwD with valid number of EDA***	43	115	158	
PwD with 1 admission, n (%)	33 (76.7%)	81 (70.4%)	114 (72.2%)	
PwD with 2 or more admissions, n (%)	10 (23.3%)	34 (29.6%)	44 (27.8%)	
Rate of EDA per PwD/year	0.26	0.43	0.37	
PwD with at least 1 HA (with > 24 h stay), n (%)	58 (9.0%)	135 (12.8%)	193 (11.4%)	0.018
PwD with valid number of HA***	57	133	190	
PwD with 1 admission, n (%)	50 (87.7%)	103 (77.4%)	153 (80.5%)	
PwD with 2 or more admissions, n (%)	7 (12.3%)	30 (22.6%)	37 (19.5%)	
Rate of HA per PwD/year	0.35	0.50	0.44	
Department of HA, n (%)				
Geriatric	5 (10%)	49 (35%)	54 (28%)	
Internal Medicine	15 (31%)	41 (29%)	56 (29%)	
Surgical	20 (41%)	20 (14%)	40 (21%)	
Other	11 (22%)	30 (21%)	41 (22%)	
Length of stay during HA admission in days, median (Q1;Q3)				
Geriatric	8 (5;16)	8 (6;11)	7.5 (6;11)	
Internal Medicine	11 (4;15)	8 (6;17)	7.5 (5;17)	
Surgical	6.5 (3;17)	8.5 (4;11)	7 (3;15)	
Other	3 (2;6)	9 (6;18)	7 (4;14)	

Note: *Q1* first quartile, *Q3* third quartile

*: PwD: People with Dementia

**: A PwD may have suffered both EDA and HA episodes

***: Data on the number of EDA or HA available only for these participants

****: *p* values provided only for the main descriptive variables on EDA and HA

Two-hundred and eighty-four of the people with dementia (16.7%) experienced at least one EDA (< 24 h) or HA between the baseline and follow-up assessments, with a higher frequency in the HC group (NH: 89 (13.8%) vs HC: 195 (18.5%); *p* = 0.011). The majority of the people with dementia living at home and in nursing home who suffered at least one EDA within the 3 months had only one episode of EDA, with a minority of them suffering two or more EDA. A similar situation occurred for HA episodes.

We found an overall estimated HA rate per person with dementia/year of 0.44 and EDA rate per person with dementia/year of 0.37, with an estimated rate of HA per person with dementia/year of 0.35 among persons living in NH and 0.50 among those living in HC, and an estimated rate of EDA per person with dementia/year of 0.26 among persons living in NH and 0.43 among those living in HC.

Some differences were found with regard to the types of departments where people with dementia were admitted, depending on their living situations.

### Factors associated with emergency department and hospital admissions

Table 4 displays the univariate and multivariate analyses on the factors potentially associated with having at least one EDA or HA for each setting. The multivariate analyses showed that, for people with dementia living in NH, polypharmacy was associated with having at least one EDA or HA (OR 1.96, CI (1.03–3.75)). With regard to the people with dementia living in HC, the factors independently associated with hospital admission were: the presence of falls in the preceding 3 months (OR 1.64, CI (1.12–2.41)), polypharmacy (OR 2.48, CI (1.70–3.62)), weight loss (OR 1.73, CI (1.17–2.56)), mild caregiver burden compared with little or no burden (OR 1.67 CI (1.04–2.66)), and moderate to severe caregiver burden compared with little or no burden (OR 1.76, CI (1.04–2.98)). No statistically significant association was found between hospital admission and functional or cognitive status of participants in either NH or HC.

**Table 4:** Univariate and multivariate analysis on the factors potentially associated with having at least one EDA or HA, by setting

	Nursing Home (***n*** = 646)					Home Care (***n*** = 1054)				
	PwD with at least one EDA or HA (***n*** = 89; 13.8%)	PwD without EDA or HA (***n*** = 557; 86.2%)	***p***_value	Unadjusted OR [95%CI]	Adjusted OR [95%CI]	PwD with at least one EDA or HA (***N*** = 195; 18.5%)	PwD without EDA or HA (***n*** = 859; 81.5%)	***p***_value	Unadjusted OR [95%CI]	Adjusted OR [95%CI]
Age (years) n (%)			0.120					0.562		
65–80 years	17 (19.1%)	151 (27.1%)		Reference	Reference	65 (33.3%)	307 (35.7%)		Reference	Reference
≥ 81 years	72 (80.9%)	406 (72.9%)		1.58 (0.90–2.76)	1.94 (0.98–3.85)	130 (66.7%)	552 (64.3%)		1.11 (0.80–1.55)	1.12 (0.78–1.61)
Sex, n (%)			0.085					0.282		
Female	60 (67.4%)	426 (76.5%)		Reference	Reference	119 (61.0%)	560 (65.2%)		Reference	Reference
Male	29 (32.6%)	131 (23.5%)		1.57 (0.97–2.55)	1.55 (0.88–2.75)	76 (39.0%)	299 (34.8%)		1.20 (0.87–1.65)	1.06 (0.74–1.52)
S-MMSE score, n (%),^a^			0,954					0,175		
Mild dementia	8 (12.5%)	58 (12.8%)		Reference	Reference	42 (24.6%)	198 (26.3%)		Reference	Reference
Moderate dementia	37 (57.8%)	242 (53.3%)		1.11 (0.49–2.51)	1.00 (0.42–2.38)	81 (47.4%)	396 (52.6%)		0,96 (0,64-1,45)	0.93 (0.60–1.45)
Severe dementia	19 (29.7%)	154 (33.9%)		0.89 (0.37–2.16)	1.13 (0.46–2.78)	48 (28.1%)	159 (21.1%)		1.42 (0.90–2.26)	1.3 (0.78–2.17)
Weight loss, n (%)			0.724					0.002		
No	80 (89.0%)	485 (86.6%)		Reference	Reference	140 (70.9%)	703 (81.4%)		Reference	Reference
Yes	9 (11.0%)	72 (13.4%)		0.80 (0.38–1.67)	0.72 (0.32–1.62)	55 (29.1%)	156 (18.6%)		1.79 (1.25–2.57)	1.73 (1.17–2.56)
Polypharmacy, n (%)			0.049					< 0.001		
No	20 (22.5%)	186 (33.4%)		Reference	Reference	51 (26.2%)	399 (46.4%)		Reference	Reference
Yes	69 (77.5%)	371 (66.6%)		1.73 (1.02–2.93)	1.96 (1.03–3.75)	144 (73.8%)	460 (53.6%)		2.45 (1.73–3.46)	2.48 (1.70–3.62)
Falls in preceding 3 months n (%)			0.009					0.001		
No	55 (61.4%)	419 (75.1%)		Reference	Reference	137 (70.1%)	702 (81.7%)		Reference	Reference
Yes	34 (38.6%)	138 (24.9%)		1.90 (1.19–3.05)	1.66 (0.96–2.86)	58 (29.9%)	157 (18.3%)		1.91 (1.34–2.71)	1.64 (1.12–2.41)
Charlson Comorbidity Index n (%)			0.906					0.076		
≤ 2	55 (61.8%)	349 (62.7%)		Reference	Reference	131 (67.2%)	632 (73.6%)		Reference	Reference
> 2	34 (38.2%)	208 (37.3%)		1.04 (0.65–1,64)	1.04 (0.61–1.77)	64 (32.8%)	227 (26.4%)		1.36 (0.97–1.90)	1.13 (0.78–1.64)
Katz Index, n (%)			0.221					0.029		
Severe	60 (67.4%)	327 (58.9%)		2.94 (0.69–12.58)	2.00 (0.44–9.04)	84 (43.3%)	281 (33.1%)		1.60 (1.01–2.54)	1.24 (0.72–2.15)
Moderate	27 (30.3%)	196 (35.3%)		2.20 (0.50–9.72)	1.96 (0.43–8.97)	80 (41.2%)	407 (47.9%)		1.06 (0.67–1.67)	0.95 (0.57–1.58)
Mild	2 (2.2%)	32 (5.8%)		Reference	Reference	30 (15.5%)	161 (19.0%)		Reference	Reference
NPI-Q Severity, mean (SD)	7.7 (6.0)	6.8 (5.7)	0.174	1.03 (1.00–1.07)	1.02 (0.97–1.07)	9.9 (6.1)	9.0 (6.2)	0.069	1.02 (1.00–1.05)	0.99 (0.96–1.02)
Zarit Burden Index n (%)			0.511					0.018		
Little or no burden	47 (53.4%)	264 (47.6%)		Reference	Reference	37 (19.0%)	227 (26.5%)		Reference	Reference
Mild burden	28 (31.8%)	211 (38.0%)		0.75 (0.45–1.23)	0.6 (0.34–1.1)	88 (45.1%)	398 (46,5%)		1.36 (0.89–2.06)	1.67 (1.04–2.66)
Moderate to severe burden	13 (14.8%)	80 (14.4%)		0.91 (0.47–1.77)	1.00 (0.47–2.15)	70 (35.9%)	231 (27.0%)		1.86 (1.20–2.88)	1.76 (1.04–2.98)

*CI* Confidence Interval, *EDA* Emergency Department Admission, *HA* Hospital Admission, *OR* Odds Ratio, *PwD* People with Dementia

^a^ Mild Dementia (S-MMSE score > 21), Moderate Dementia (S-MMSE score 10–21), or Severe Dementia (S-MMSE score < 10 or not evaluated due to impaired cognitive or functional status)

### Costs of emergency department and hospital admissions

Table 5 shows the estimated health cost per person with dementia/year according to country and setting (NH vs HC). On average, for the whole study sample, the estimated medical cost per person with dementia/year related to EDA and HA was 1884.28€, with 1046.19€ per person with dementia/year in NH group and 2397.96€ in HC group. Concerning the estimated average expenditure per person with dementia/year for EDA, this was 67.30€ in the NH group and 117.48€ in the HC group. For HA an estimated average cost per person with dementia/year of 978.89€ was obtained in the NH group, and 2280.48€ in the HC group. On average, for the whole study sample, we found higher expenses per person with dementia/year in both ED and HA visits for people with dementia living in the HC setting compared to those living in the NH setting, except in the Netherlands, where a higher expenditure on hospitalisation of people with dementia living in NH was found, despite the global EDA and HA cost per person with dementia/year being lower than the HC group. A greater estimated cost per person with dementia/year for EDA was also found in the NH group in France and Germany.

**Table 5:** Estimated average annual medical costs per person with dementia in the RTPC sample (*n* = 1700) ^a, b,c^

Medical Care (Inpatient care)	Estonia ***n*** = 225	Finland ***n*** = 268	France ***n*** = 186	Germany ***n*** = 190	Netherlands ***n*** = 269	Spain ***n*** = 240	Sweden ***n*** = 207	UK ***n*** = 115	All countries ***n*** = 1700
**Estimated n of people with dementia** **with EDA episodes** **(estimated EDA episodes/year)**	48 (56)	124 (164)	56 (68)	84 (108)	72 (144)	136 (200)	36 (108)	76 (96)	632 (944)
**Cost (NH)**	6.31	62.89	122.78	121.75	51.77	52.51	35.77	127.96	67.30
**Cost (HC)**	20.87	183.41	68.30	105.96	135.17	115.19	169.51	164.21	117.48
**Total cost of EDA**	15.69	133.49	78.55	114.19	102.93	92.99	119.76	146.87	98.41
**Estimated n of people with dementia** **with HA episodes** **(estimated HA days/year)**	72 (816)	148 (2656)	176 (1832)	140 (1072)	44 (204)	56 (1012)	56 (1308)	72 (1000)	764 (9900)
**Cost (NH)**	215.86	1092.88	1326.99	1464.11	353.11	397.94	2005.59	1229.82	978.89
**Cost (HC)**	459.23	5340.47	3632.84	2429.68	153.14	1372.76	3028.60	1619.27	2280.48
**Total cost of HA**	372.70	3581.21	3198.94	1926.57	238.17	1059.53	2648.13	1433.01	1785.87
**Estimated n of people with dementia** **with hospital episodes (EDA episodes and HA episodes)**	120	272	232	224	116	192	92	148	1396
**Cost (NH)**	222.17	1155.77	1449.77	1585.86	424.83	540.84	2041.56	1357.78	1046.19
**Cost (HC)**	480.10	5523.87	3701.13	2535.65	288.31	1487.96	3198.11	1783.48	2397.96
**Total cost of hospital episodes**	388.39	3714.70	3277.49	2040.76	341.09	1152.52	2767.89	1579.89	1884.28

*EDA* Emergency Department Admissions, *HA* Hospital Admissions

^a^All expenses refer to costs per person with dementia, according to setting and country

^b^Costs calculated including only people with dementia with data available on the number of EDA or HA

^c^All costs expressed in €

## Discussion

In this cross-sectional study, 1700 people with dementia from eight European countries participating in the RTPC study were investigated. The estimated rate of hospital admission per person with dementia/year of 0.35 among people living in NH and 0.50 among those living in HC, and an estimated rate of emergency department admission per person with dementia/year of 0.26 among people living in NH and 0.43 among those living in HC. In the HC setting, factors identified as being associated with a higher probability of suffering at least one EDA or HA were: suffering weight loss, accidental falls, presenting polypharmacy and having a caregiver with mild to severe burden compared with little or no burden. In the NH setting, only polypharmacy was associated with a higher probability of suffering at least one EDA or HA. Concerning health costs, a higher estimated cost per person/year attributable to hospital admission (EDA and HA) was observed for people with dementia included in the HC group as compared to the NH group.

With regard to community-dwelling people with dementia, our results of estimated rate of hospitalisation are within the range of hospitalization rates presented by Shepherd et al. in their systematic review of the literature (rates of 0.37–1.26 per person/year) [9]. When comparing our results on hospitalisation rates among people with dementia living in NH, the few studies existing for this setting report on disparing results, with Maxwell et al. (2018) reporting a rate of 0.21 person/year [10], and Feng et al. (2014) reporting a rate of 0.46 [39].

People with dementia living at their homes had higher rates of EDA or HA than those in the NH setting in the present study. Reasons for this may have been that caregivers of people with dementia who live at home may have had less support in managing health needs or higher levels of care burden [40], leading to a greater need for emergency service utilization and hospital admission. Other reasons for lower hospital transfer needs in the nursing home setting compared with home care suggested in the literature are that, even if EDA rates from nursing home are high [8], some of these institutions may be equipped with specific care units for people with advanced dementia [41], have a wider availability to perform post-acute attention [42], receive specialized health support from geriatric [43] or end-of-life care more frequently, and they may have policies to prevent transferring those persons to hospital.

Sleeman et al. also found in a retrospective cohort study of 4867 people with dementia in their last year of life in England, that being in a nursing home was associated with a significantly lower number of ED visits [43]. The authors argued that increasing bed capacity in NHs may have explained their results, as it may have allowed more people with advanced dementia dying in the NH.

Maxwell et al. also reported on lower hospitalisation rates among people with dementia living in NH compared with those living in assisted living facilities, and attributed this difference to the insufficient capacity of assisted living facilities to cover the needs of people with dementia [10].

Conversely, another report analyzing the impact of dementia on hospital and ED use among fee-for-service Medicare beneficiaries reported that people with dementia who lived in nursing homes were significantly more likely to visit the emergency department and be hospitalised than those who resided in their own homes [29].

In the HC setting, polypharmacy was associated with hospital admission, in the line with the results by Shepherd et al., who found moderate confidence in this association in their meta-analysis [9]. In contrast, Shepherd et al. found moderate confidence in the association of lower level of functional ability with hospitalisation risk. This association was not found in the present study. Instead, in the present study significant associations were found with a history of falls within the previous 3 months and with the presence of weight loss. These factors were not evaluated by Shepherd et al. in their meta-analyses. Interestingly, the authors suggest in their discussion that it can be difficult to ascertain whether loss of functionality may be connected with other adverse outcomes such as side effects of medication or even malnutrition or weight loss. This might explain the differences between their results and those of the present study.

So far, unintentional weight loss has been found associated with increased morbidity and mortality among older adults [44], but to the best of our knowledge the relationship between unintentional weight loss in people with dementia and the risk of hospitalisation has not been well established [9, 45]. In previous reports, but in older populations, malnutrition has been associated with an almost fourfold increased risk of readmission or mortality within 7 days of discharge from a hospital, and the risk almost doubled between 8 and 180 days after discharge [46].

Conversely, falls are of great concern for people with dementia given their potentially higher number of visits to emergency departments and severe consequences, mainly hip fracture [47]. Our results are aligned with those reported by Toot et al. on the risk of admission associated with osteoarticular conditions (eg: falls/fractures) in people with dementia living in the community [48].

The severity of dementia was not associated with hospital admission, which is consistent with the results presented by Shepherd et al. in their meta-analyses, who reported moderate confidence in this finding [9].

Conversely, higher comorbidity was not associated with hospital admission in the present study, in contrast with the results by Shepherd et al., who graded their confidence with this result as moderate. However, in their discussion, the authors reported on a study [49] that described an increased risk of hospitalisation only because of the presence of dementia as comorbidity, irrespective of the existence of any further comorbid conditions.

Currently, evidence for effective interventions to reduce the risk of falls, to reduce polypharmacy and to improve nutrition is limited and requires further development [50–53]. Nevertheless, the results of the present study suggest that the identification and management of these three geriatric syndromes might help preventing hospitalisation events among people with dementia living at home. Furthermore, the promotion of an advanced and individualised care plan among people with dementia showing these geriatric syndromes may be particularly advisable.

Mild to severe levels of caregiver burden were associated with hospital admission for people with dementia living in the HC setting in the present study. This result is in the line with the results by Guterman et al. [14] and Maust et al. [40], which found a significant association between mental health and caregiver distress, respectively, and ED use by people with dementia living in the community. Future interventions to reduce unnecessary hospitalisations should also focus on providing support to the caregivers of people with dementia living at home.

In the NH setting, only polypharmacy was associated with hospital admission among people with dementia in the present study, suggesting that there is a special need to target this geriatric syndrome for this population, in order to reduce unnecessary hospitalisations [54, 55].

In the present study, it was observed that medical costs associated with hospital admission (including EDA) were higher in the HC setting, results that can be explained by the higher frequency of HA and EDA found among people with dementia living at home. Similar to our results, Coots Daras et al. (2017) found that people with dementia in their last year of life living in NH had lower costs derived of EDA and HA than people with dementia living in the community. However, these results were not seen among the general population with dementia, for whom the costs of EDA and HA were higher among those living in NH [16]. The authors discuss that expenditures associated with hospital and ED use may vary by residential setting, and proximity to death.

Our results suggest that interventions aimed at preventing EDA and HA among people with dementia may be cost-effective, especially in the HC setting, as for most countries the costs associated with EDA and HA were higher in these setting. However, the results should be interpreted with caution, as costs of other interventions that may be put in place when acute events occur such as specialized health support teams or specific care units for advanced dementia have not been taken into consideration. Differences between countries with regard to the implementation of such interventions, as well as potential country differences in the characteristics of the people with dementia living in each of the settings [56] and potential country differences in the way how dementia care is provided [57], may explain the country differences seen in the costs of hospitalisation. Furthermore, these results should be seen within the framework of the overall costs of dementia care. Interestingly, a previous analysis of the costs of dementia care within the RTPC study showed higher costs for the care provided at NH compared with those provided at HC, with the particularity that the costs in the HC setting increased with the severity of dementia [33].

The findings of our study should be interpreted considering its limitations. Firstly, there might have been a number of people with undiagnosed dementia who were not included in the present study due to its inclusion criteria (formal diagnosis of dementia); these people, who may also be admitted to hospital for several reasons with the corresponding costs associated, could not be described in the present study. Secondly, the time period investigated for each participant was limited to 3 months, which may not represent the entire population of people with dementia requiring hospital admission over the one-year period, taking into account seasonal fluctuations; however, the prospective character of the study may have facilitated the acquisition of reliable data. Furthermore, the RTPC country samples may not be representative of the general national population of people with dementia. Thirdly, although we accounted for multiple variables that may affect the need for hospital admission, there may also be additional unmeasured confounding factors that influence the use of hospital care, for example, specific health support at the community or nursing home setting in each country or even at the local level. Finally, several years have passed between data compilation and secondary analysis. However, we believe that the present study adds relevant value to the body of literature with regard to the comparison of the frequency of hospitalisation between the NH and HC settings among people with dementia, factors associated and costs, in a world where the organization of long-term-care is still a challenge [58].

## Conclusions

To the best of our knowledge, this is the first study that evaluates internationally (across Europe) the frequency of hospitalisation among people with dementia living in nursing homes and home care settings, and analyses the associated factors and costs for each setting.

Admission to hospital is frequent among people with dementia, especially among those living in the community, and seems to impose a remarkable economic burden. The identification and establishment of an individualized care plan for those people with dementia with polypharmacy in nursing homes, and those with involuntary weight loss, accidental falls, polypharmacy and higher caregiver burden in the home care setting, might help preventing unnecessary hospital admissions and saving associated costs.

## Supplementary information

**Additional file 1: ** Baseline characteristics of informal caregivers.

## Data Availability

The datasets used and/or analysed during the current study are available from the corresponding author on reasonable request.

## Abbreviations

CCI: Charlson Comorbidity Index
COI: Cost-of-illness
EDA: Emergency Department Admission
€: Euros
EU: European Union
HA: Hospital Admission
IEA: International Epidemiological Association
KATZ: Katz Index of Independence in Activities in Daily Living
NH: Nursing Home
NPI-Q: Neuropsychiatric Inventory-Questionnaire
OECD: Organisation for Economic Co-operation and Development
OR: Odds Ratio
PPP: Purchasing Power Parities
Q1: Quartile 1
Q2: Quartile 2
RTPC: RightTimePlaceCare
RUD: Resource Utilization in Dementia
SD: Standard Deviation
S-MMSE: Standardized Mini-Mental State Examination
WHO: World Health Organization
ZBI: Zarit Burden Interview
